# Complexities in supportive care for people with metastatic breast cancer: a qualitative study

**DOI:** 10.1007/s11764-024-01646-8

**Published:** 2024-08-17

**Authors:** Marika Franklin, Sophie Lewis, Andrea L. Smith

**Affiliations:** 1 School of Health Sciences, The University of Sydney, Sydney, Australia; 2 The Daffodil Centre, The University of Sydney (a joint venture with Cancer Council NSW), Sydney, Australia; 3 School of Nursing & Midwifery, The University of Sydney, Sydney, Australia

**Keywords:** Supportive care, Metastatic breast cancer, Health and community-care professionals, Patient-professional relationships, Ethics of care, Qualitative research

## Abstract

**Purpose:**

The complexity of metastatic breast cancer, its rapidly evolving treatment, and the changing trajectory toward long-term survivorship create unique challenges for the provision of supportive care. The experiences of health professionals enacting supportive care in contexts of those living long-term with incurable cancer have received limited research attention. This qualitative study aimed to gain further insight into health professionals’ experiences of supportive care in this context.

**Method:**

Semi-structured interviews were conducted via phone and online with 25 health and community-care professionals who support people living with metastatic breast cancer in Australia. A mix of sampling strategies was used. Thematic analysis was undertaken. Findings were interpreted through an ethics of care lens.

**Results:**

Three key themes were identified. First, participants experienced supportive care as highly relational. Second, they encountered numerous moral and ethical dilemmas in enacting supportive care. Finally, enacting supportive care was complicated by fragmented and sporadic provision in a system in which supportive care is differentially valued across professions and settings.

**Conclusion:**

Findings draw attention to complexities in enacting supportive care in the context of metastatic breast cancer, with implications to patients and professionals. To improve the quality of care provided to patients and minimise the risk of professional burnout, greater attention is needed in supportive care guidelines to the ethical, moral, and emotional complexities experienced by professionals in this context.

**Implications for Cancer Survivors:**

People living with metastatic breast cancer are a growing proportion of cancer survivors. The knowledge gained through this study may help professionals to better meet the supportive care needs of people living with metastatic breast cancer, a treatable but not curable condition.

## Introduction

Rapid advances in cancer treatment, such as targeted therapies and immunotherapies, are changing the life course of many people diagnosed with metastatic breast cancer. As these individuals move toward longer-term survivorship, their support care needs are becoming increasingly complex [1–3]. While considerable gains have been made in prognosis, metastatic breast cancer remains incurable for almost all patients [4]. Metastatic breast cancer is the second leading cause of cancer death globally in women [5] and of the approximately 600,000 deaths from breast cancer globally each year, the vast majority are due to metastatic breast cancer [6]. Treatment options and outcomes vary according to the breast cancer subtype and patient characteristics, resulting in complex and rarely predictable or linear disease trajectories [7–9]. People with metastatic breast cancer can experience various medical, emotional, and existential challenges [1]. This unpredictability and complexity are significant challenges for the provision of care, including both how professionals conceptualise and enact supportive care. In this article, we aim to examine the experiences of health and community-care professionals who care for and support people with metastatic breast cancer, to gain a better understanding of the complexities of supportive care in this context.

The Multinational Association of Supportive Care in Cancer defines supportive care as the prevention and management of the symptoms and side effects of cancer and its treatment across the cancer continuum from diagnosis to end of life including support for patients, their families, and their caregivers [10]. National and international guidelines recognise the importance of integrating appropriate supportive care as a routine part of cancer care for people living with metastatic cancer [10–12]. Furthermore, supportive care is promoted as being person-centred, holistic, and responsive to individuals’ multidimensional care needs across the entire cancer trajectory. The multidimensionality of supportive care needs for people with metastatic breast cancer involves a range of professionals in clinical and non-clinical settings [10, 13–15].

Variations in how supportive care is conceptualised occur throughout the literature [15–17]. There is also limited clarity around the roles and responsibilities of different health and community-care professionals (e.g. who should lead care/provide services) and how resources should be allocated [15, 17]. Evidence indicates that supportive care services primarily focus on the initial diagnosis and treatment phases and end-of-life care [18]. Furthermore, evidence shows care pathways, traditionally designed as optimal, linear pathways to make the disease more navigable, have not been adequately adapted to reflect the complexity, variability, and precariousness of conditions such as advanced cancer [19]. For instance, supportive care services are often fragmented, and oncologists often refer patients to other services/professionals (e.g. social workers, pain management, psychologists, and palliative care) in an ad hoc way [15, 19, 20]. These issues can lead to delayed referrals, and unequal access and use of supportive care services for people with metastatic breast cancer [15]. They also contribute to higher reported unmet supportive care needs for patients living with treatable but not curable cancers [1].

The issues with fragmentation of supportive care are further compounded by Australia’s complex healthcare landscape that includes both public and private services. Medicare, Australia’s universal public health scheme, provides free or subsidised health to all citizens, supplemented by private services through opt-in private health insurance or full-fee user pay options. Australia also operates a Pharmaceutical Benefits Scheme (PBS) that subsidises some pharmaceutical treatments but not others. While advances in treatment have created new outlooks for patients with metastatic breast cancer, unless listed on the PBS the high cost of some of the newer treatments can leave some people substantially out of pocket, and some may not be able to access them at all [1, 18]. Similarly, access and cost of supportive care services vary widely. The inequalities in the affordability and access to treatments and supportive care services for people with metastatic cancer impose new ethical and moral responsibilities on policymakers and health professionals, especially as more people are living with treatable, but not curable cancer, and living longer.

### A practice-based approach to supportive care

Despite the imperative of supporting people to live with cancer (and not merely to recover or die from) [18], there has been limited research on supportive care for those living with incurable cancers see, for exception [15, 21]. Here, we examine health and community-care professionals’ experiences of providing care and support for people living with incurable breast cancer (herein as metastatic breast cancer), with a particular focus on the challenges and dynamics of enacting ‘good’ care. We draw on an ethics of care approach, viewing care as inherently relational, not solely a practice of giving care to another, but also associated with caring *with* others, accentuating the interdependency of care [22–24]. From this perspective, care involves maintaining relationships and harm avoidance, and empathetic situated and contextual decision-making from multiple perspectives [23, 25, 26].

Understanding care as situated and relational means that we cannot take care and the way it is enacted for granted, as the meanings of care and complexities of practicing care are entwined with specific norms, values, and contexts [23]. Accordingly, what one context considers ‘good’ care, may be ‘bad’ care in another. For instance, care that benefits the care-receiver may cause harm to the caregiver and vice versa. Moreover, different types of ‘good’ and/or ‘bad’ care can co-exist [23]. Different logics of care such as professional logics (e.g. treatment of patients according to standardised evidence-based guidelines) versus relational logics (e.g. meaningful interconnections between people) can lead to different ethical concerns and understandings of what constitutes ‘good’ care.

In this study, we seek to reveal the ethical, moral, and emotional complexities health and community-care professionals may grapple with when enacting supportive care for patients with metastatic breast cancer. We use an ethics of care lens to examine the logic underpinning professionals’ practices in supportive care. The concept of ‘logic’ enables us to examine the patterns of practice which make the everyday supportive care practices of these professionals understandable and sometimes even ‘natural’ to those involved [23, 27]. While we use the concept of logic to acknowledge what makes the supportive care actions of health and community-care professionals appear reasonable and appropriate, we do not imply that these practices will be coherent and logical [27]. Thus, the purpose of this research is to take a closer look at how supportive care for people with metastatic breast cancer occurs in practice. This knowledge will guide future developments in the care and support of people living with metastatic breast cancer in Australia.

## Methods

### Study design

As we were interested in exploring subjective experiences and how participants gave meaning to their experiences, we were guided by a social constructionist approach to qualitative research [28]. Data were drawn from semi-structured interviews with 25 participants. The interviews were conducted as part of a larger research project which also investigated Australian women’s experiences of metastatic breast cancer and cancer care. Ethics approval was granted bythe University of New South Wales Human Research Ethics Committee. University Human Research Ethics Committee blinded for review].

### Participants and recruitment

Eligible participants were health and community-care professionals who were providing care and/or support for patients with metastatic breast cancer. Due to the relatively small number of health professionals working with people with metastatic breast cancer, a mix of purposive [29] and snowballing sampling [29], and community-based [30] recruitment strategies were employed. This mix of strategies enabled the recruitment of a diverse range of health and community-care professionals from various disciplines working across hospital and community-based settings who could provide relevant and in-depth insights about supportive care for people with metastatic breast cancer. Participants were recruited via flyers, advertisements, and presentations through cancer organisations and peak bodies; email invitations direct to health professionals whose contact details were publicly available, snowball recruitment through study participants, and through diverse relationships established by the research team with other community partners such as organisations providing wellness programs and support groups for women living with metastatic breast cancer. Through these various strategies, potential participants were provided with contact details of a member of the research team, who they were invited to contact to express their interest. Examination of demographic characteristics (including professional type, years working in the cancer field, and setting type) throughout the recruitment process indicated that we had recruited participants with diverse experiences. We note that four professionals who expressed an interest in the study did not proceed to an interview for reasons unexplained when contacted via email to arrange an interview.

### Data collection

The interviews were semi-structured, using an interview guide. Aligned with the social constructionist approach, the interview guide was developed in recognition that that there are multiple ways of understanding ‘care’. The interview guide was informed by a review of the literature, discussions with the broader project team, and consultation with a consumer representative with metastatic breast cancer. The data collection phase was open and sensitive to capturing the alternative views health professionals may hold of ‘care’ in relation to the phenomena of supportive care. Topics explored included challenges and facilitators to support patients with treatable but not curable cancer, positive and negative aspects of one’s role, and perceptions of what constitutes supportive care. Two qualitative researchers, who are both female and experienced in undertaking research on sensitive topics, conducted the interviews. After informed consent was obtained either in writing or verbally, interviews were conducted in person, over telephone, or video conferencing, according to the preferences of the participant. Interviews were conducted between 2019 and 2020 and ranged from 35 to 98 min (mean = 59 min; median = 61 min). The interviews were audio-recorded and later transcribed in full and de-identified.

### Data analysis

Drawing on an interpretive approach, thematic analysis [31] was used to understand health and community-care professionals’ perceptions and experiences of supportive care. The process of analysis was deductive and inductive, and throughout analysis, consideration was given to the significance and meanings that health and community-care professionals ascribed to ‘care’ in supportive care for people with metastatic breast cancer. Conceptually, our analysis was informed by the ethics of care scholarship including the work of Held [22] which emphasises care is both a practice and a value, shaped by moral understandings infused with emotions. Care is also relational and interdependent. This lens enabled us to examine how values, moral reasoning, and affective responses shape supportive care in the context of metastatic breast cancer. An ethics of care is situated within social constructivism that centralises a person-centred approach to care.

Coding commenced following the first interview, enabling refinement of prompts for questions in subsequent interviews. Initial coding of the transcripts was conducted in Nvivo 11 by the first author, who closely and repeatedly read each transcript, made detailed notes, and developed codes. Codes were compared within and across transcripts to identify similarities and differences, with categories developed from which broader themes encapsulating health and community-care professionals’ perceptions of rewarding and challenging aspects of providing supportive care and how these aspects of their work are managed personally and/or professionally. This process was conducted through discussions among the authors until any differences were resolved [32]. This led to the development of three key themes: (1) the relationality of supportive care; (2) moral and ethical dilemmas of enacting care; and (3) ‘caring well’ is constricted by a fragmented system.

The three authors involved in the analysis are female academics, from diverse disciplines (sociology and social and health sciences), with different experiences of metastatic breast cancer, and one with living experience. All are experienced qualitative researchers whose research focuses on lived experiences of long-term conditions and how experiences are shaped by social context. The inter-disciplinarity of the research team and variations in experiences ensured that the analysis and interpretation of data were undertaken from multiple perspectives and not from any one health profession or discipline. Throughout the study design, data collection, and data analysis process, the authors reflected and engaged in discussions on how their research experiences, disciplinary backgrounds, and experiences with metastatic breast cancer were shaping the interpretation of the data. This process involved the authors critically examining their personal characteristics and perspectives in relation to the participants, the meanings of supportive care and care more broadly.

## Findings

In total, 25 participants, from 18 different organisations, were interviewed. For six of these organisations, two or three participants, in different positions or with different years of experience were interviewed. Participants comprised breast care nurses (*n* = 9), support group facilitators (*n* = 4), alternative and complementary medicine practitioners (*n* = 4), specialist clinicians (*n* = 3), support service managers/coordinators (*n* = 3), and allied health professionals (*n* = 2). Fourteen participants worked in hospital settings and 11 were from non-hospital settings. Participants were also diverse in terms of the number of years working in the advanced cancer space, settings in which they worked including public and private hospitals, private practice and not-for-profit community organisations, and geographical location (metro/regional across four Australian States). All participants were women.

### The relationality of supportive care

The first theme developed in our analysis focused on the relationality of supportive care. Participant accounts focused on the interconnectedness of supportive care between themselves and the person with metastatic breast cancer. Here, participants emphasised relations of responsibility, reciprocity, care, and friendship. They did not see themselves as solely a provider of care. When they described their caregiving role, they emphasised the privilege of walking ‘beside someone’ and what they learned from patients with metastatic breast cancer about what it means to live authentically. These aspects of their role reinforced their sense of being in a personally fulfilling and privileged role. The following extract exemplifies these elements of reciprocity, of give and take of supportive care:

They’re living with this uncertainty all the time. And so, they’re very real, they’re very present. They don’t beat around the bush, there’s no sort of bullshit about them, and it’s just really refreshing to be in their presence […] it’s just a real privilege. (Mia, nurse)

Participants also characterised their role as honourable work. Supporting a person throughout a particularly emotionally difficult and vulnerable time in their life, often over an extended period, from their diagnosis with metastatic cancer to their death, was constructed by participants as highly valuable and important work. The honourable nature and relationality of care with these patients were further evident in the emphasis participants placed on the close and meaningful interpersonal relationships formed with patients with metastatic breast cancer. These relationships were described as ‘special’, ‘profound’, ‘important’, and ‘unique’ for patients, their family members, and health professionals alike. The following extract from a clinician illustrates the depth to which health professionals get to know patients with metastatic breast cancer and their families over time:

…you know them very well because you’ve looked after them for 10 years, or 20 years sometimes, since they were first diagnosed, and so they and their families are really very real and important people to interact with, rather than someone that you’ve met for 10 minutes. (Hana, clinician)

In a similar vein, a nurse participant discussed the privilege of being a confidante for a person with metastatic breast cancer, providing them with a safe space to vocalise feelings and thoughts that were taboo in their interactions with their family, friends, and other health professionals:

I meet them at one of their most vulnerable times, so they do open-up and talk and it’s just a real privilege to be let into that. (Mia, nurse).

From participant accounts, it was clear that they felt a deep sense of care and responsibility for the well-being of patients with metastatic breast cancer and in doing so struggled at times to maintain their own sense of well-being. While participants described their roles as personally fulfilling, the enduring and intimate nature of the interactions and relationships with patients with metastatic breast cancer often came with a sense of responsibility to these patients that could be emotionally taxing. Participants described the pressure they felt in maintaining authentic and contextualised relationships with these patients, to not let them down, and to ensure that these patients were well supported. Sustaining the emotional and physical investment that was required as people with metastatic breast cancer can live for extended periods of time took its toll on participants. A narrative of ‘enduring’ was often described, as one participant put it: ‘you’ve got to keep up your stamina for the marathon’ (Hana, clinician).

The intimate nature of relationships and connections between professionals and patients with metastatic breast cancer impacted on how participants managed patient-professional boundaries. One nurse reflected on the grief and emotional toll that she experienced when a particular patient with metastatic breast cancer died. For this nurse, forming an authentic relationship with the patient and their family was central to accomplishing ‘good’ care, but it also meant the boundaries of such a relationship became blurred. She articulates the difficulty in balancing genuine human connections (relational logics) with the pressures to maintain professional boundaries (professional logics) and care for self:

We had a patient who I got very close to. I did her eulogy at her funeral, I became friends with her […] The consequence of that is that I get affected by that. I do miss her and I still speak to her husband and her sisters. I knew the whole entire family. Her mum and dad, like she was 35 […] Yeah, it’s really only to my own detriment [...] because I’m the one who then gets upset about it […] but we are humans at the end of the day. (Raelene, nurse)

The impact of burnout for those working in the field of incurable cancer was also raised by participants. Participants’ accounts highlight the tricky balance between caring well for those with metastatic breast cancer, while at the same time preserving personal well-being and mental health. Sharing the load and working as a team was seen as imperative to minimise burnout:

Because you cannot do this role and carry that level of burden. You’ve got to be able to step away and access other services and pull in things, because the longevity in this role for any worker would be so less and the risk of burnout would be so high if you didn’t access all other services and work as a team to get the outcome for these patients. (Amanda, nurse) 

However, many expressed a lack of support and expressed a desire for formalised support to assist health and community-care professionals in managing their own well-being.

I think there needs to be professional support, because it is a really tough job. So, I think there needs to be some professional support… there’s not formal support (Raelene, nurse)

### Moral and ethical dilemmas of enacting supportive care

A second prominent theme generated through the analysis was how to manage moral and ethical dilemmas while still providing good supportive care for people with metastatic breast cancer. Participants’ accounts revealed how their perceptions of ‘good’ care were subject to (re)negotiations of their core values, moral reasoning, and affective responses in relation to the logics of biomedicine, professionalism, and person-centredness. The complexities of these dynamics were evident in participants’ descriptions of trying to balance both hope and realism in their interactions with patients with metastatic breast cancer. The dialectic of hope/realism is illustrated by the following nurse, who talked about the challenge of preparing someone for death, while also giving them hope about their future:

… the bit I find really difficult is trying to maintain some hope knowing that they probably have only got 12 months or two years to live. So, wanting them to be prepared and prepare the family and their will and all of that sort of stuff, but also not wanting to take away their hopes. So, I find that’s always a tricky conversation. (Mia, nurse)

Similarly, the following excerpt shows how the desire to provide comfort and optimism to patients with metastatic breast cancer and to avoid evoking additional distress and pain, could also get in the way of what participants deemed as necessary conversations (e.g. about palliative care and advance care planning, death and dying). Moreover, the absence of these hard, but necessary conversations could close off opportunities for health and community-care professionals to facilitate access to supportive care services (e.g. palliative care services). When and how to have these conversations were also complicated by treatment advances contributing to periods of disease stability and extended survival for people living with incurable cancer. In the following extract, a clinician draws attention to how seeing a patient with metastatic breast cancer do well over an extended period of time can create a false sense of security for the patient, as well as the health professional regarding the realities of living with an incurable condition.

And sometimes as a doctor it can also be hard because you’re getting this false sense of security when someone’s doing well for a long time and perhaps you don’t have those conversations as often as you should. So, I guess it’s just trying to sort of keep reminding people what’s happening. But at the same time, you don’t want to be seeing them every time and saying, “You’re dying” and destroying all hope. (Monica, clinician)

The constraints of the normative logic of cancer (e.g. around cure and death) differentially impacted the way in which participants engaged in supportive care. The following nurse’s quote illustrates her feeling of personal responsibility to challenge some of these normative logics of cancer (e.g. the pursuit of cure and imperative of positivity) to provide the patient and their family with information that will allow them to plan and prepare appropriately. Her excerpt also highlights the difficulties encountered in providing care as part of an interdisciplinary team, when professionals from different disciplinary backgrounds were pursuing different (and sometimes competing) goals, priorities, expectations, or values.

So, they [oncologists] tend to be almost eternally optimistic to the point of palliative care sometimes isn’t always discussed, and that’s more concerning, I think, to be honest, where the actual subject isn’t brought up…So that there’s a time for that client to access palliative care and have all the conversations and prepare, is shortened because of that. So, that is concerning. (Rebecca, nurse)

This extract from Rebecca reveals how the unfolding of different logics of care, and power differentials within, give rise to different ethics. For Rebecca, her description of the oncologists’ care practices is counterintuitive to the patients’ needs, rights, choices, and freedoms. For Rebecca, the actions of the oncologists amount to *poor* rather than *good* care, amounting to what she sees as an injustice in the patients’ right for earlier supportive care.

A second moral challenge participants described was the tension of respecting patient autonomy and choice versus their desire to provide good care. This tension is reflective of challenges arising for health and community-care professionals when key principles of person-centred care become misaligned or out of step. Illustrative of how this tension impacts health and community-care professionals, the following is a quote from a nurse participant who described her pain and sense of helplessness from being a spectator to a patient who opted out of mainstream cancer treatment and experienced in her words, ‘awful deaths’. Her focus on young mothers also illustrates how the impacts of bearing witness to a patient’s suffering were tied to broader cultural and moral questions, in this case, expectations about motherhood and caring responsibilities. Furthermore, this example shows how inter-dependencies and vulnerabilities can circulate both inwards and outwards of the patient-health professional dyad:

Young women who decide not to have mainstream treatment, who decide to go alternative and decide to use really unproven fasting diets or extreme diets or some particular cure cancer diet that they’ve found on the internet, I do struggle with that a lot because I’ve had a couple of women, two women anyway, who have done that and died pretty awful deaths and left really young children. So, I’ve struggled with that. (Mia, nurse)

Finally, participants talked about providing supportive care for patients who were experiencing financial difficulties. They particularly spoke about issues surrounding access to clinical trials and treatment options that were financially prohibitive (e.g. those that were not publicly subsidised) for patients with limited financial resources. The following nurse spoke of the internal ethical conflict which arises when offering patients treatments that are financially burdensome or prohibitive, and the extraordinary out-of-pocket costs that patients are required to pay for these potentially life-extending drugs. Moreover, this nurse draws attention to the inequity in access to cancer treatment.

I have so many people that are just struggling to pay for their food at the checkout and keep a roof over their head, let alone then pay for expensive drugs. That’s really, really difficult. And it’s something I find really hard, that if you have more money, you get certain drugs that may not be available for people that can’t afford them. It’s pretty awful, really. (Olivia, nurse)

Similarly, another participant draws attention to inequities in the financial burden for people with metastatic breast cancer associated with accessing clinical trials, particularly for people living outside of metropolitan areas where major cancer centres are located. Here, the participant highlights additional costs related to travel and accommodation for people needing to travel to access the clinical trial, as well as the personal cost of being away from work and family:

I think there’s much less access for patients because they’ve got to be able to travel and they’ve got to travel quite often. Some women will be seeking that because they hear of a [Name of Cancer Centre] and they want to access it and they choose to do it. But then they can be travelling. They could be much more isolated from work or family or whatever because they’re travelling more often. It can be quite difficult for them to do that. (Sonya, nurse)

Furthermore, the uncertainty as to whether high-cost interventions are worth the cost for people with metastatic breast cancer in terms of years lived, but also quality of life was also said to complicate the support health and community-care professionals could provide around financial management and treatment decisions. Further drawing attention to these issues, another nurse commented:

I had a particular lady who was paying $5,000 a month for these tablets, and she said, “Look, I’m running out of money.” And I said, “I know. I know. I’m trying.” Because she said, “How long am I’m going to be on it for?” I said, “I don’t know.” It’s a double-edged sword. I want her to be on it for as long as possible because it means it’s working and it’s agreeing with her, but no one’s got a bottomless pit of money. (Mia, nurse)

Participants also drew attention to the difficulty they and patients with metastatic breast cancer experienced in trying to access support to relieve financial pressures. The following quote by a support service program coordinator emphasises the hard work and burden experienced in providing this aspect of supportive care:

…navigating the whole maze of welfare stuff, of Centrelink, of disability pensions, of where you can get support. I think that’s just in the woods as well really. I feel like there is possibly some support out there, but you have to work so hard to find it and get to it and apply with it that that’s a real burden sometimes. (Hazel, support service program coordinator)

The following extracts highlight equity issues related to accessing expensive drugs on compassionate grounds. This access was linked to relationships between health professionals and drug companies, a drug company’s willingness to provide compassionate access, and professionals’ experience in navigating compassionate requests:

Because I have a lot to do with the sort of top end of the drug companies, because of being involved in clinical trials, I normally have someone on speed dial that I can ask and that’s helpful. So, I’d say to them, “Look, this guy owes me a favour,” or I already know that they are not giving any away. (Hana, clinician)

And that was really hard trying to find out if they could get it on compassionate grounds or whether there was some other way he could get it, or he could get it for his wife, at least. And you feel really helpless because often there aren’t any other avenues. So yeah, I do struggle with that a bit. (Mia, nurse)

When speaking of instances of healthcare inequity, participants expressed a deep sense of personal responsibility and duty and often went above and beyond to attend to their patients’ unique situation to make up for the lack of resources or supports that was provided for people with metastatic breast cancer. In this way, health and community-care professionals sought to take accountability for the social and economic context in which they operate to minimise its impact on people with metastatic breast cancer. For instance, the following community-based nurse talked about reusing lymphedema sleeves for their patients who could not afford them:

That’s another large group that sort of misses out on stuff. They’re falling through the cracks. I would ask my clients to please give me their old sleeves, because there would be people out there that can’t afford a sleeve, and that’s not on Medicare. There’s no medication that will help you with your lymphedema. And so, there’s nothing on the PBS [pharmaceutical benefits scheme] and it’s a lifelong issue. (Heather, nurse)

### ‘Caring well’ is constricted by a fragmented system

Participants described the challenge of working within a healthcare system where supportive care services for people with metastatic breast cancer were often positioned as an ‘add-on’ rather than integrated into cancer care. In general, participants described supportive care as not well integrated, patchy, disjointed, and/or undervalued. All participants commented on the challenges of navigating fragmented, siloed, and complex systems for both professionals and patients with metastatic breast cancer (e.g. difficulties sharing information across services, time-limited appointments, and patients needing to attend different clinics at different times).

… there’s no regular pathway or discussion about, “How’s Mrs so and so doing psychosocially?” That there is no MDT [multidisciplinary team] that looks at anything other than their clinical needs. […] But again, it’s a resourcing issue that there isn’t time for that sort of thing. So that’s a big frustration. (Grace, nurse)

Many participants reported a lack of access to supportive care providers such as psychologists, social workers, allied health workers, and palliative care providers. As such, as the following clinician articulates, the burden to fill supportive care gaps tended to fall on the oncology team (e.g. oncologists, oncology and metastatic breast care nurses):

We have a shortage of psychologists and breast care nurses and people that can offer a lot of that extra support. So, often it does fall on us, as oncologists, to sort of do a lot of that psychologist, palliative care professional stuff. You’re sort of doing a lot of those roles yourself, often because it’s hard to get access to all the allied health that you need. (Monica, clinician)

Some participants, particularly those who worked in the community setting, rather than in a hospital, perceived that their expertise in providing supportive care was unrecognised or undervalued by professionals working in hospital settings. Participants described the perceived lack of recognition for the positive contribution that they could make as difficult. As the following participant, a nurse working in the community, describes, while one of the goals of supportive care is working together as part of an interdisciplinary team, some professions or roles were excluded or institutionally devalued within existing models of supportive care.

…sometimes it’s challenging when, say, other health professionals might not treat me in a respectful way. So, because we work in a charity, sometimes we’re not on the frontline, so people don’t always understand how we work, where we fit in, and our worthiness. (Rebecca, nurse)

Another participant, a psychologist and support group facilitator working in a hospital, described how some aspects of supportive care, especially the focus on psycho-social wellbeing, are devalued in clinical settings. This devaluing was linked to the dominant clinical focus in education and training for some professions which orient practice toward cure *over* care, rather than having these go hand in hand:

Right through the whole system, the psychosocial will come bottom of the pile […] and lack of respect for a non-clinical role when you’re in the clinical environment […] As much as I love working with nurses and doctors, they come from a different training and background […] So there’s less respect for a steady psycho-social approach. (Olivia, allied health professional)

A negative consequence of the lack of an integrated approach of supportive care within cancer care was that patients with metastatic breast cancer were said to be ‘falling through the cracks’ and not getting the care that they needed. Participants expressed feelings of frustration and helplessness about the limits of the system, which constrained supportive care for these patients. When expressing feelings of frustration, participants also revealed their own vulnerabilities. For example, a complementary and alternative medicine practitioner describes the sense of frustration and fragility experienced in struggling to meet the complex needs of a patient with metastatic breast cancer that were not met through the supportive care available in the hospital system:

People […] are being let down by the system. She just had a lot of medical needs. And she’d been turned away from the hospital, and I was her only hope, and I couldn’t help. So, there’s times that it’s quite heartbreaking. (Jessica, complementary and alternative medicine practitioner)

Throughout the interviews, participants emphasised the complex nature of the clinical and supportive care needs for women living with metastatic breast cancer and the difficulties they face in meeting these needs in a healthcare system they perceived to be ill-equipped to support these complexities. Furthermore, many participants indicated that the impact this has on health and community-care professionals working in this space is not being attended to.

## Discussion

This study provides rich, theoretically informed insights into how diverse professionals working in advanced cancer care conceptualise and enact supportive care. Through examining professionals’ subjective experience of supportive care through an ethics of care lens, our study reveals how the relationality of supportive care gives rise to different ethical, moral, and affective dilemmas in this context. Navigating these dilemmas requires professionals to engage in complex (re)negotiations around care relationships, and how care is delivered and evaluated. These revelations make two main contributions to understanding the complexities of supportive care for people with metastatic breast cancer.

First, for health and community-care professionals in this study, supportive care was highly relational. It involved tenuous negotiations on how to manage social bonds, interdependencies, and various relations between patients with metastatic breast cancer and professionals, and amongst professionals themselves. Participants considered the relationship between patient and professionals and mutual displays of trust, respect, authenticity, and vulnerability between professionals and patients with metastatic breast cancer as constitutive of *good* quality supportive care. They emphasised that providing *good* care involved paying close attention to the specificities of the person and the totality of the persons’ experience of cancer. They also emphasised what they had learned from their patients, and how these experiences had enriched their professional and personal lives. In this way, they brought to the forefront the humanity of patients with metastatic breast cancer, as well as their own humanity and the intertwined and mutual nature of care [22]. Relationships such as these are shown to be more possible in less biomedical contexts and to also allow for and promote ‘agency, self-determination and ultimately hope’ [33], p. 43.

Interdependence is not only recognised and valued according to relational logic, but also as giving rise to discomfort and vulnerability see also [22, 24]. The vulnerability of health and community-care professionals and patients with metastatic breast cancer was evident throughout our findings. For instance, participants perceived that they must foster meaningful and authentic relationships to care well, but they also experienced this as emotionally and physically taxing. Furthermore, participants expressed their frustration and sometimes felt helpless because of constraints in the healthcare system they worked in, and the unpredictable and progressive nature of the disease itself, often limited their ability to provide *good* supportive care. Some lamented that despite their best efforts, they could not do enough for their patients. Additionally, tension was evident for participants between a desire on the one hand to provide a person-centred, holistic approach to care, versus on the other hand, maintaining professional and relational boundaries. Others have also reported on the difficulties health professionals can have in maintaining boundaries in caring relationships, particularly in the context of incurability or end-of-life [34].

A second and related finding from our study was that enacting supportive care was challenging due to how supportive care and those who provide supportive care, are valued, and embedded in cancer care and the healthcare system more broadly. Like previous supportive care research, our findings showed that health workforce shortages, lack of funding, and fragmentation of care were all obstacles to the provision of supportive care for people with metastatic breast cancer [21, 35]. Additionally, some participants, particularly those working outside of the hospital setting or in non-clinical positions, described experiencing difficulties associated with establishing their place within a wider network of professionals involved in the care of patients with metastatic breast cancer, and how others valued the care they provided. These participants highlighted the influence medical and professional logics have on the prioritisation of treatment and cure over psychosocial needs, contributing to psychosocial aspects of care being under-recognised and undervalued by some health professionals, and within the healthcare system more broadly. This led to variations amongst professionals in when and how topics were raised with patients, what options and information were presented to patients, and the extent to which they focused on people’s clinical, physical, practical, or psychosocial needs. They discussed these issues in terms of ethical and moral dilemmas, such as balancing the imperatives of positivity and hope with authenticity and realism but they also related them to concerns of injustice see also [36].

Participants articulated a particular moral struggle was the experience of supporting patients with metastatic breast cancer when life-extending treatment and services to increase the quality of life were inaccessible due to external factors such as access to clinical trials, compassionate access, or an (in)ability to pay rather than individual needs see also [37]. High levels of uncertainty in the length of time someone living with advanced cancer may need treatment and the effectiveness of the treatment also compounded this issue see also [38]. Some of these moral and ethical issues stem from the increasingly blurred categorisation of conditions such as metastatic breast cancer as terminal or chronic, and the lack of understanding that for metastatic breast cancer (and other treatable but not curable cancers) this is not a dichotomy but that both apply. The way that health and community-care professionals, patients, and institutions categorise incurable conditions can give rise to different values and meanings for when and how long to give life-extending treatment alongside/or versus the prioritisation of supportive care needs see also [39, 40], p.1. We recommend further research to examine how competing values and meanings shape care for people with advanced cancer and how these issues impact on patients’ and professionals’ well-being.

A key strength of this study is the provision of in-depth and theoretically informed insights into the practice of *care* in the contemporary context of supportive care from the perspective of a diverse range of professionals. These findings highlight the intertwined nature of an affective orientation of care (i.e. caring about) and the practical/logistical side (i.e. doing care) of supportive care work in the context of a treatable but not curable condition. It also points to various vulnerabilities for health and community-care professionals due to constraints on enacting supportive care in a way which aligns with their perceptions of what it means to care and support someone to live ‘well’. The issue of health professional vulnerability is receiving increasing attention in the oncological and palliative care literature, through issues such as compassion fatigue e.g. [41], moral distress e.g. [42], and burnout e.g. [41, 43–45]. Our study suggests that further investigation of these issues is warranted in the context of supportive care for people living with incurable conditions, who are *in between* cure and end of life.

The limitations of this study also need to be noted. Although this study was diverse in terms of professionals, setting type, and years working in supportive cancer care, the experiences of some health and community-care professionals working with patients with metastatic breast cancer may not have been captured. Particularly, the study was conducted in Australia, and the sample consisted of females only. Different findings may be found amongst a mixed gender or male sample, and in different geographical contexts, with different health systems. Furthermore, due to the small number of different types of health and community-care professionals in this study, conclusions specific to types of professionals or settings are limited. As the meanings and values placed on supportive care, resource constraints and inequities vary across settings and in relation to different types and stages of cancer, further research will assist with understanding the nuances within supportive care and implications for health and community-care professionals’ well-being.

## Conclusion

These findings show that new roadmaps or guides are needed for supportive care to better align with the extended nature of the cancer trajectory for people with metastatic breast cancer. These roadmaps need to include guidance for health professionals on how to manage the ethical and moral dilemmas arising in supportive care for people with metastatic breast cancer. Moreover, with treatment advances extending the length of time some patients are living with incurability and increasing the range of professionals and settings involved in care provision, clarity is needed on who is and should be part of multidisciplinary teams for people living with advanced cancer and when and what this involvement looks like [18, 19]. Additionally, in line with an ethics of care approach, it is recommended that the wellbeing of health and community-care professionals be part of any new roadmap for supportive care. We recommend further research to direct the development of these roadmaps and guidelines.
